# Mycosynthesis of Metal-Containing Nanoparticles—Fungal Metal Resistance and Mechanisms of Synthesis

**DOI:** 10.3390/ijms232214084

**Published:** 2022-11-15

**Authors:** Martin Šebesta, Hana Vojtková, Veronika Cyprichová, Avinash P. Ingle, Martin Urík, Marek Kolenčík

**Affiliations:** 1 Institute of Laboratory Research on Geomaterials, Faculty of Natural Sciences, Comenius University in Bratislava, Ilkovičova 6, 841 04 Bratislava, Slovakia; 2 Department of Environmental Engineering, Faculty of Mining and Geology, VŠB—Technical University of Ostrava, 17. Listopadu 2172/15, 708 00 Ostrava-Poruba, Czech Republic; 3 Biotechnology Centre, Department of Agricultural Botany, Dr. Panjabrao Deshmukh Agricultural University, Akola 444 104, India; 4 Department of Soil Science and Geology, Institute of Agronomic Sciences, Faculty of Agrobiology and Food Resources, Slovak University of Agriculture in Nitra, Tr. A. Hlinku 2, 949 76 Nitra, Slovakia

**Keywords:** biosynthesis, green synthesis, nanomaterial, metallic nanoparticle, metal oxide nanoparticle, fungus, biomolecule

## Abstract

In the 21st century, nanomaterials play an increasingly important role in our lives with applications in many sectors, including agriculture, biomedicine, and biosensors. Over the last two decades, extensive research has been conducted to find ways to synthesise nanoparticles (NPs) via mediation with fungi or fungal extracts. Mycosynthesis can potentially be an energy-efficient, highly adjustable, environmentally benign alternative to conventional physico-chemical procedures. This review investigates the role of metal toxicity in fungi on cell growth and biochemical levels, and how their strategies of resistance, i.e., metal chelation, biomineral formation, biosorption, bioaccumulation, compartmentalisation, and efflux of metals from cells, contribute to the synthesis of metal-containing NPs used in different applications, e.g., biomedical, antimicrobial, catalytic, biosensing, and precision agriculture. The role of different synthesis conditions, including that of fungal biomolecules serving as nucleation centres or templates for NP synthesis, reducing agents, or capping agents in the synthesis process, is also discussed. The authors believe that future studies need to focus on the mechanism of NP synthesis, as well as on the influence of such conditions as pH, temperature, biomass, the concentration of the precursors, and volume of the fungal extracts on the efficiency of the mycosynthesis of NPs.

## 1. Introduction

In the last few decades, nanoparticles (NPs) have been increasingly used in a wide range of applications [1], such as precision agriculture and food production [2,3,4,5], biological imaging [6], catalysis [7,8], environmental remediation [9], optoelectronics [10], magneto-optics [11], personal care products [12], antimicrobiotics and pharmacology [13], etc. Chemical and physical methods of NP synthesis are the most widespread despite the major issues concerning their safety and reliable, environmentally friendly, economical way of creating them. Some of the currently employed methods are ball-milling, etching, laser ablation, spray pyrolysis, chemical vapour deposition, hydrothermal synthesis, sol-gel synthesis, micro-emulsion methods, and hybrid methods including electrochemical, photochemical, biohydrothermal, or a combination of the aforementioned routes [14]. They are potentially hazardous and require toxic chemicals and high temperatures, pH, and pressure for synthesis. Moreover, these methods involve high energy consumption and are expensive. To overcome these inefficiencies, several environmentally benign ways of NP synthesis were developed that use non-hazardous chemicals and ambient conditions, e.g., room temperature, atmospheric pressure and circumneutral pH [15,16,17]. Methods using plants, fungi, microorganisms, or their extracts were developed that fit the above-mentioned criteria of a more environmentally benign synthesis of NPs [18]. Mycosynthesis of metal-containing NPs is often preferred because of the many behaviours observed in soil formation processes that also involve the biogenic formation of minerals by fungi [15,19].

A variety of fungi can be easily grown because they are chemo-organotrophs with simple nutritional requirements and ease of manipulation with the produced biomass [19]. They can also withstand a wider range of growth conditions compared to other microbes and plants. In their natural environment, they mostly gather their food from decomposing organic matter. Even though most species of fungi are decomposers and helpful in the cycle of nutrients, there are many species that are also parasitic and pose a threat to other organisms in the environment. However, all of them secrete enzymes and other substances to digest the food extracellularly and the predigested food is then absorbed and the digestion completed internally. Thus, it is easy to collect these biomolecules necessary for NP synthesis and the NPs can be produced outside the cell walls, which makes their separation from the biomass a simple process [19]. However, some NPs were created on the cell walls, even on the inner side of the cell walls [20]. Fungi are also known to produce biogenic organic minerals in soils, such as oxalates and carbonates [21,22]. They are generally considered an important contributor to the biotransformation of soil minerals [23]. Since they facilitate weathering processes of soils, sediments, rocks, and their physical and chemical decomposition, there have been plans to use them to extract valuable elements, e.g., heavy metals, precious metals, and rare earth elements, in processes called fungal-assisted biohydrometallurgy [24].

The first scientific articles concerned with the mycosynthesis of NPs started to appear in 2001 [20,25]. In slightly more than two decades, NPs of different chemical compositions, sizes, and shapes were synthesised with the help of living fungi and their extracellular metabolites [15,26,27]. Based on chemical composition, elemental NPs, such as metal Ag, Au, Cu, Pb, Pt, and bimetallic Ag-Au NPs were created. Oxides, such as BaTiO_3_, Bi_2_O_3_, CoFe_2_O_4_, Co_3_O_4_, Fe_2_O_3_, Fe_3_O_4_, NiO, TiO_2_, ZnO, and ZrO_2_, were also synthesised. Even quantum dot NPs, CdS and CdSe, were manufactured [5,26,28]. These nanoparticles were used in various fields, i.e., in biomedicine, antimicrobial applications, catalysis, biosensing, mosquito control, and precision agriculture. As mentioned above, two pathways are used in the fungi-assisted synthesis of NPs—intracellular and extracellular [26]. The selection of a pathway depends on the species of the fungus. Often in species where intracellular pathways play a role, species-specific active sites on cell walls, plasma cell membranes and other membranes inside the fungal cell may be important for NP synthesis [15,25]. Extracellular synthesis of NPs by fungi is governed by reactions with several types of biomolecules. NADH-dependent reductases [29], nitrate-dependent reductases, electron shuttle quinones [30], and small molecules (less than 3 kDa) such as amino acids, cofactors, and glucose-based substances [31] may mediate fungal NP synthesis. Moreover, metabolites produced by fungi also play an important role as capping agents that stabilise the NPs and prevent their aggregation.

In the last few years, numerous reviews concerning the biosynthesis of metal-containing NPs were published [32,33,34,35,36,37,38,39,40,41,42,43,44,45,46,47], many specialising in either one or two types of NPs [48,49,50,51,52,53,54,55,56] or biosynthesis for special applications [57,58,59,60,61,62,63,64,65]. Among these reviews, several specialised in fungal biosynthesis—the mycosynthesis of NPs [15,26,27,66,67,68,69,70,71]. The presented reviews’ novelty and focus are on the mechanisms of mycosynthesis of several metal-containing NPs and how these mechanisms are related to the fungal resistance to metals. Metal resistance of fungi towards NPs and NP-forming elements is discussed, together with their ability to solubilise, form biogenic minerals, and transform inorganic (nano)particles in their natural environment. Moreover, the influence of different types of biomolecules and biological cell structures of fungi on NP formation is discussed. Fungus-assisted synthesis of NPs may be an environment-friendly, low-cost way of synthesising NPs that can be used in precision agriculture, medicine, antimicrobial agents, catalysis, biosensing or many other important applications.

## 2. Toxic Effects of Metals and Metal-Based NPs on Fungi

Fungi developed pathways to use metals as well as control their concentrations through evolution and interaction with the natural environment. For hundreds of years, they have also been exposed to various human activities that increased the concentrations of these elements in the environment, with the culmination of the intensity of these activities in the last two centuries. The fungi have interacted with effluents from industries, such as agricultural, automobile, fossil fuel, mining, tanning, etc. Whether the metals are deposited through air or water, soils are one of the main sinks for these metal elements, and since they are not biodegradable, they accumulate in soils where fungi interact with the increased metal concentrations [72]. The high amounts of these elements are an influential stress factor for the soil fungi, inhibiting their growth and disrupting multiple fungi–plant symbiotic interactions, which leads to decreased fertility of soils and increased accumulation of metals in plants with various negative effects [73]. Increased concentrations of metal cause cell membrane damage, organelle damage, and lipid peroxidation through processes related to the excessive generation of reactive oxygen species, which leads to cell apoptosis [74]. These stress pressures have been highly selective, and in areas with high concentrations of metals, metal-resistant fungal strains can be easily found. The fungal defence mechanism can be divided into two general categories: extracellular and intracellular strategies. Extracellular strategies inhibit the uptake and internalisation of metal ions and include such processes as the production of extracellular metabolites, which immobilise the metal ions into biogenic minerals, and the thickening of the cell wall. Intracellular strategies include the compartmentation of the metals into vacuoles, their intracellular precipitations, phosphatisation, or mineral formation with oxalates, and chelation and active transport outside the cells [26].

### 2.1. Effects on Cell Growth

High concentrations of metal ions are toxic and inhibit the life processes of fungi, with high enough concentrations leading to growth inhibition and death. Many of the fungi that synthesise inorganic NPs are inhibited by the high concentrations of metals that the NPs are made of. The metal ions, such as Cd, Cr, Cu, Ni, and Pb, have an inhibitory effect on fungi at concentrations between 0.1 mM to 100 mM [75,76,77]. The metal tolerance is both species- and strain-dependent [78]. For example, the growth of *Aspergillus biennis* was fully inhibited at concentrations higher than 10 mM of Pb [75]. Other species of *Aspergillus* in a study by Liaquat et al. [76] showed 100% inhibition at higher concentrations, i.e., 14 to 24 mM of Pb. Cadmium was found to inhibit growth by 50% at 0.2 mM for basidiomycotan fungus of *Schizophyllum commune* [79]. Typically, higher molar concentrations are needed for essential elements such as Cu and Ni, where 100% inhibition concentrations were 63.0 to 78.7 mM and 38.4 to 76.7 mM, respectively, for three species of *Aspergillus* [77].

Some metal-containing NPs have shown lower inhibition effects than their ionic counterparts. For example, when ionic-form AgNO_3_ and Ag NPs were used to stop the growth of plant-pathogenic fungi, *Bipolaris sorokiniana* and *Magnaporthe grisea*, the ionic form showed a greater reduction in colony formation [80]. On the other hand, ZnO and Cu NPs have been more effective against seven species of plant-pathogenic fungi than their ionic ZnSO_4_ and CuOH counterparts, whereas CuO NPs have shown a lower effect [81]. Released ions from NP often do not play the most important role in the toxicological effect on fungi; however, the importance of released ions is based on the chemical and crystal structure of the NPs and the surrounding environment, which at times contribute significantly to the toxicity [82,83].

### 2.2. Effects on a Biochemical Level

High concentrations of metals inhibit various parts of fungal metabolism and disrupt many common biochemical processes. Among observed effects are the high production of reactive oxygen species that lead to the depletion of antioxidants; alteration and inhibition of enzyme activity; deterioration of cellular membranes and disruption of electron and ion pumps; negative changes in nutrient uptake; and conformational changes in nucleic acids and proteins that disturbed transcriptional and translational steps of gene expression [84,85]. The exact pathways of inhibition are specific for each element. For example, Ni alters carbohydrate metabolism, resulting in the release of pyruvate and the disintegration of membrane structures in *Neurospora crasa* [86]. Cadmium, a non-essential element for fungi, is highly toxic even in low concentrations, and toxic concentrations increased the production of proteins, lipids, and carbohydrates, whereas activities of enzymes like lipases, amylases, and proteases were highly reduced in ascomycotan fungi, such as *Aspergillus carbonarius* and a strain of *Penicillium* sp. [87]. It probably displaces Zn and Ca from zinc finger proteins and metalloproteins [88,89]. Yeast *Saccharomyces cerevisiae* that was exposed to high concentrations of Cd led to hypermutability, which was a result of inhibiting post-replication mismatch repair of DNA [90]. Methylmercury inhibits the activity of some enzymes, e.g., L-glutamine:d-fructose-6-phosphate amidotransferase, in yeasts [91]. In addition, heavy metals, such as Cd^2+^, Hg^2+^, and Pb^2+^, interfere with the refolding of chemically denaturated proteins [92]. Both Cd and Cr(VI) cause the accumulation of aggregated proteins in the cells of yeast [85]. Moreover, at toxic concentrations, most of the essential and non-essential metals increase oxidative stress by the creation of reactive oxygen species in fungal cells, which is one of the most influential reasons for growth inhibition and death of fungi [93,94].

Depending on their chemistry, crystallinity, size, and shape, NPs have been shown to exert various negative effects on fungi. Their toxic effect may come from two main effects: the release of ions at toxic levels that disturb the homeostasis of fungal cells and the direct interaction of NP surfaces with fungal cells leading to membrane damage, disruption in biochemical processes in cells, and reactive oxygen species generation [95]. Even though NPs exert toxic effects, they have been found to have relatively low toxicity. In single cellular fungus, yeast *S. cerevisiae,* NPs of Al_2_O_3_, CeO_2_, Fe_2_O_3_, HfO_2_, TiO_2_, and ZrO_2_ did not inhibit O_2_ uptake and showed negligible membrane damage at concentrations as high as 1000 mg∙L^−1^, when the yeast was grown in bioassay medium. Their low toxicity was due to the formation of settleable micron-sized agglomerates [96,97]. On the other hand, Mn_2_O_3_ NP reduced O_2_ uptake by 50% in *S. cerevisiae* at 170 mg∙L^−1^ and caused up to 30% cell membrane damage at higher concentrations. Fe(0) NPs showed only low toxicity to *S. cerevisiae* [97]. Size-related toxicity of NPs to *S. cerevisiae* was shown with PbS NPs, and the toxicity increased with decreasing size. The NPs damage the cell wall of *S. cerevisiae,* while the defensive response of the yeast enhances production of chitin and genes responsible for cell wall integrity signalling are overexpressed. In addition, intracellular levels of reactive oxygen species increased, leading to mitochondrial dysfunction and cell apoptosis (Sun et al., 2014). Ag NPs induced antioxidant enzyme activities in two strains of an ascomycete—aquatic fungus *Articulospora tetracladia*. Gene ontology enrichment analysis also showed that enzymes that had functions in DNA repair and energy production were induced. Both Ag NPs and ionic Ag^+^ induced proteins related to ascospore formation, cell redox and protein homeostasis, fatty acid biosynthesis and nucleic acid metabolism, as well as stress-responsive proteins [82].

## 3. Metal Resistance in Fungi

**Figure 1:**
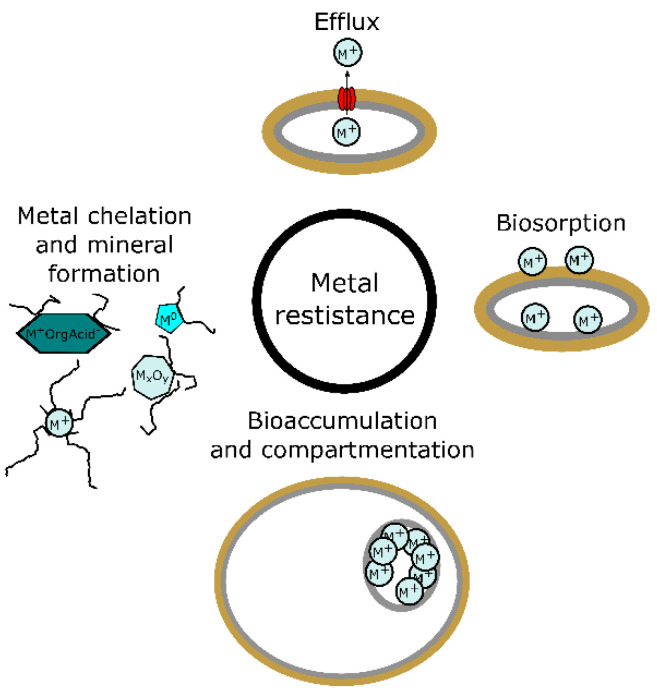
Strategies of metal resistance in fungi.

Resistance of fungi to metal toxicity is the result of the long evolutionary process. Fungi and their ancestors have been exposed to various concentrations of metals in the environment for the whole existence of life on Earth. That led to the development of strategies that involve mechanisms that lower the concentrations of metals getting into fungal cells, lower the toxicity of metals in the intracellular environment and help with the efflux of excessive amounts of metal from the inner cell environment. These strategies can be divided into four related categories (Figure 1): (1) metal chelation and intra- and extracellular mineral formation, (2) biosorption, (3) bioaccumulation and compartmentation, and (4) efflux of metal, which are presented later in the text. The resistance processes on cellular and molecular levels are studied to the largest extent in the model organism, ascomycete yeast *Saccharomyces cerevisiae*, and substantial knowledge of the metal resistance strategies has been gained on ascomycete filamentous fungi and also in basidiomycetes, the mushrooms. However, some detailed knowledge is still missing [93]. Metal resistance is an important property that predisposes certain genera or species of fungi to be better suited for bio-utilisation in the synthesis of metal-containing NPs [26]. Some of the most resistant genera to metal toxicity were found to be *Aspergillus* sp. and *Penicillium* sp. [98]. Most of the time, fungal strains isolated from contaminated sites have higher tolerances [99]; however, there were also a few cases [100], such as Cd-resistant *Piptoporus betulinus*, where there was no found relation between increased resistance and isolation from contaminated and uncontaminated sites [101].

### 3.1. Metal Chelation and Intra- and Extracellular Mineral Formation

**Figure 2:**
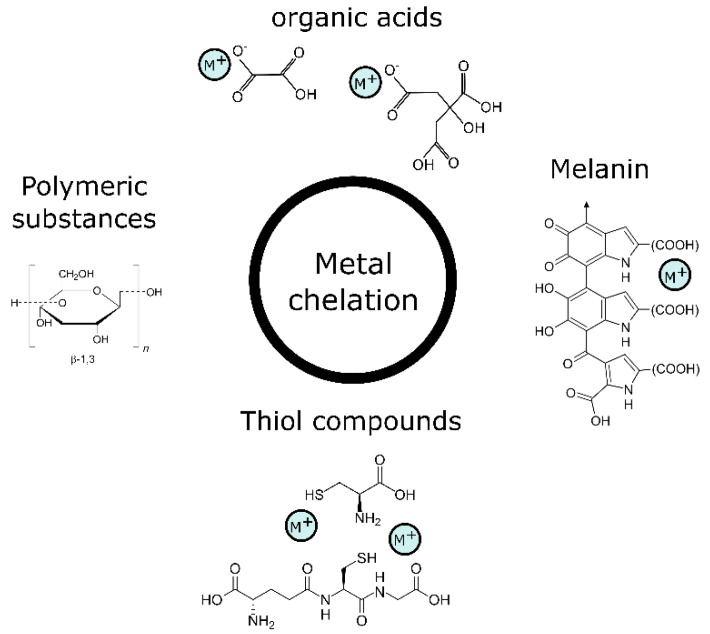
Metal chelation compounds.

Fungi produce a large variety of organic molecules that they release into the environment to gather important organic and inorganic nutrients, and also to protect themselves from the harsh conditions of the outside environment, such as elevated concentrations of metals. This gives them the ability to decrease the toxicity of these metals. Furthermore, it can lead to the formation of biogenic minerals that lower bioavailable concentrations in the vicinity of fungi. Fungi can produce high amounts of metal chelators, which they use to maintain their cell homeostasis and decrease the toxicity of the metals. The chelators are released both intra- and extracellularly and help with extracellular mineral formation, intracellular compartmentalisation and the efflux of metals back to the external environment of the fungal cells. Various organic compounds, such as thiol-functionalised molecules, metallothioneins, homogeneous and heterogeneous proteins, peroxidases and similar enzymes, organic acids, and biopolymers (Figure 2), play a role in the precipitation or detoxification of metals in the outer and inner environments of fungi [102,103].

#### 3.1.1. Thiol-Containing Compounds

Among the many different chelating compounds, several peptides that contain thiol groups were observed to help with metal resistance in fungi [102]. Reduced glutathione is involved as an antioxidant in the detoxification of H_2_O_2_ and O_2_ caused by Cd in the basidiomycete *Paxillus involutus* [104]. Increased concentrations of glutathione and g-glutamylcysteine were also observed by Courbot et al. [105] when the ectomycorrhizal fungus *P. involutus* was exposed to Cd. Similarly, reduced glutathione is produced in higher concentrations during toxic Cd exposure by the endophytic fungus *Piriformospora indica* [106].

Metallothioneins are low-molecular-weight, cysteine-rich proteins that are able to bind essential and non-essential metals mainly through their thiol group and are produced by fungi, algae, and plants in response to metal stress. Their involvement in Cu and Zn resistance in fungi is long known and was reported in the basidiomycete *Pisolithus tinctorius* as far back as 1986 [107]. Resistance to Zn was observed in the basidiomycete *Russula atropurpurea* and the protective actions were attributed to the activity of cysteine-containing peptides RaZBP1 and RaZBP2. RaZBP shares 77% similarity with metallothionein, and Zn binds with cysteine and histidine parts of the RaZBP molecules [108].

#### 3.1.2. Polymeric Substances

Fungal polymeric substances were found to have large sorption area and many active sites that help to alleviate metal toxicity. Special attention has been paid to extracellular polymeric substances that are comprised of polysaccharides, proteins, nucleic acids, lipids, uronic acid, and other minor organic and inorganic compounds [109]. There are several characteristics of extracellular polymeric substances that play a role in their ability to adsorb metals. A large number of carboxyl groups and hydroxyl groups, mainly contained on the surfaces of proteins, were responsible for the high biosorption of Cd^2+^, Pb^2+^, and Zn^2+^ [110]. The extracellular polymeric substances contribute largely to the ability of fungi to sorb metals and thus alleviate their negative effects. In the high metal ion-tolerant fungal strain of *Aspergillus niger* [111], it was observed that extracellular polymeric substances contribute to a large extent to the tolerance and biosorption of Pb^2+^. Moreover, the amphiphilic nature of the polymeric substances also played a role, since metal ions are also captured by electrostatic attraction and ion exchange. The content of proteins in polymeric substances plays a large role in the biosorption of metals [110]. However, there has been a report on higher sorption of Zn by polycarbonate fraction of polymeric substances [112]. Extracellular polymeric substances act as a template for the adsorption of metal cations to which carbonate ions are attracted which induces local supersaturation and formation of a new biogenic solid phase. The presence of extracellular polymeric substances promotes the formation of rounded, smoothed crystals or spheroids and formation of crystal polymorphs [113]. Wood-rotting fungi *Daedalea quercina*, *Phanerochaete chrysosporium*, *Pleurotus ostreatus*, and *Schizophyllum commune* produce polymeric substances, Cu-bio-ligands, to reduce Cu toxicity. These were in the size range of 20 to 60 kDa in *P. chrysosporium*, *P. ostreatus*, and *S. commune*, and *D. quercina* produced polymeric substances with lower molecular weights. In addition, in *P. chrysosporium*, the change in the functional groups was observed in Cu-stressed fungi compared to the control [114]. Extracellular polymeric substances of *P. chrysosporium* were also tested for immobilisation of Pb. Soluble extracellular polymeric substances had lower sorption capacity compared to bounded extracellular polymeric substances. The increased amount of polysaccharides was probably responsible for the better immobilisation of Pb, and Pb formed precipitates on the bounded extracellular polymeric substances [115]. Suh et al. [116] found that the extraction of extracellular polymeric substances helped Pb to penetrate the cell interior, confirming their important role in the extracellular capture of metal ions. Changes in the ratio of proteins and polysaccharides in extracellular polymeric substances produced by *P. chrysosporium* were recorded [117] when the fungus was grown with sublethal concentrations of Ni^2+^. A higher concentration of proteins was observed with a lower concentration of polycarbonates, which indicates the involvement of proteins in fungal tolerance to Ni^2+^.

#### 3.1.3. Melanins

Another group of chelating compounds produced by fungi are melanins. The term “melanin” encompasses a varied group of dark polymeric pigments found in all three domains of life—in bacteria, archaea, and eukaryotes—including plants, fungi, and animals. In fungi, melanin plays diverse roles in protection against different kinds of stress, including stress from ionising radiation, heat and cold shock stress, drought stress, hydrolytic enzyme stress, and stress from the accumulation of potentially toxic elements including metals [118]. The composition of melanins varies and they often contain aliphatic hydrocarbons, phenolic units, peptides and several functional groups. This provides many active sites to bind metals, and metal–melanin complexes aggregate and create granules with high electron density [119]. According to the precursor substances (used in synthetic pathways), fungal melanin can be categorised as eumelanin, 1,8-dihydroxynaphthalene melanin, 4-glutaminylhydroxybenzene melanin, pyomelanin, and pheomelanin. In addition, the useful classification of melanin is based on its location in the cell. Accordingly, melanin is classified as cell wall melanin, cytoplasmic melanin and extracellular melanin [120]. Melanin produced by ascomycetes *Aureobasidium pullulans* and *Cladosporium resinae* had a higher sorption capacity for Cu ions compared to the whole biomass of the fungi. Protection against toxicity of ionic Ag, in the form of AgNO_3_, was attributed to the generation of melanin in a human pathogenic fungus—the basidiomycete *Cryptococcus neoformans* [121]. The addition of melanin to the albino *A. pullulans* grown in the presence of Cu resulted in lower toxicity [122]. Secretion of melanin to reduce the toxicity of ionic Fe was observed in ectomycorrhizal fungi as one of the mechanisms for Fe detoxification. However, Berthelot et al. [123] found that melanin did not contribute to the metal tolerance of Cd and Zn in dark septate endophytic fungi *Leptodontidium* sp., *Cadophora* sp., and *Phialophora mustea*, and just helped with Cd accumulation. Because of their high affinity towards metals, fungal melanin has been used as recyclable biosorbent for the removal of metals (Cu^2+^, Pb^2+^, Cd^2+^, and Zn^2+^) from contaminated effluents [124]. A more detailed description of the roles of fungal melanin can be found in [120] and [118].

#### 3.1.4. Organic Acids

Several genera of fungi produce organic acids to cope with metal stress. These organic acids have the ability to solubilise metals and also form metal–organic acid precipitates, most often oxalates. These processes of metal chelation with organic acids occur in both intracellular and extracellular environments. Oxalic and citric acid production in response to metal stress was reported in many ascomycetes, including *Aspergillus* sp. and *Penicillium* sp. for metals such as Al, Co, Cu, Cd, Pb, Zn and others [21,125,126,127,128]. Fomina et al. [129] reported that oxalic and citric acids were overproduced to reduce the toxicity of metals, such as Cd, Cu, Pb, and Zn, in the entomopathogenic fungus *Beauveria caledonica*. In an adaptation study, subsequent generations of *Aspergillus foetidus* increased the production of organic acids to adapt to higher concentrations of metal ions [130]. The released oxalates may form crystals in the extracellular environments to decrease their concentrations in the environment. Such a formation was observed for Ni, where nickel oxalate dihydrate minerals were formed when a multitolerant strain of *Aspergillus niger* was exposed to high concentrations of Ni. However, no such mineral formation was observed for Cd, Co, Cu, or Cr in the same study [131]. In basidiomycetes, production of oxalic acids as a response to metal toxicity of Co and Zn was observed in species of wood-rotting fungi, such as *Bjerkandera fumosa*, *Fomitopsis pinicola*, *Phlebia radiata*, and *Trametes versicolor* [132]. In addition, *F. radiculosa* produced copper oxalate as a defence mechanism against Cu toxicity [133].

### 3.2. Biosorption

**Figure 3:**
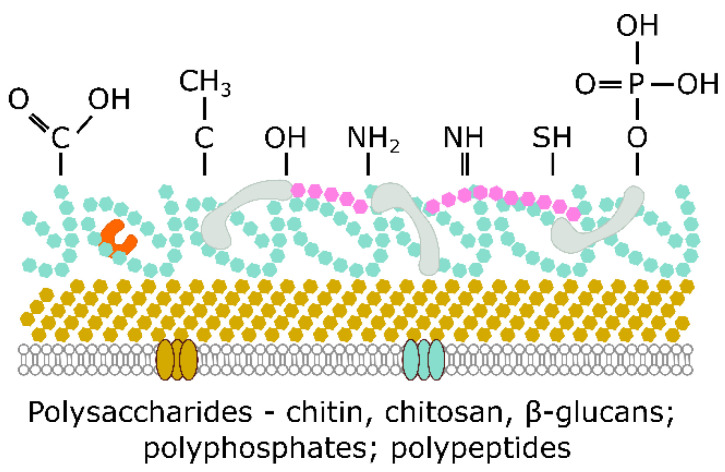
Functional groups and molecules responsible for metal sorption in fungi.

The cell wall of fungi provides a large surface for the sorption of metal ions. It has plenty of sorption sites, with most of them having a negative charge. The negative charge is a result of the many functional groups, including –COOH, –O–CH_3_, –OH, –NH_2_, =NH, –O–PO(OH)_2_, and –SH groups, on the surface of the cell wall, which is mainly made of polysaccharides such as chitin and β1,3-glucan, then proteins, polyphosphates, polypeptides, and other molecules (Figure 3). Thickening of the cell wall can thus provide fungi with a strategy that decreases the number of metal ions that enter the inner environment of their cells, preventing a toxic build-up of positively charged metal ions [134]. Filamentous fungi such as *Aspergillus* sp. and *Penicillium* sp. have been found to be a more efficient sorbent than both yeast and bacteria since their cell walls can be comprised of as much as 90% of polysaccharides with a high amount of sorption sites [135]. Amino groups of chitosan have been considered to be the primary sites for heavy metals of Cr, Cu, Ni, and Zn absorption in *Penicillium chrysogenum* [136]. *Acremonium pinkertoniae* biosorbed Cu, which resulted in the mycelium turning bluish-green. The cell wall’s glucan–chitin complex was the place of the incorporation of Cu and formation of crystalloids via bonds of Cu with amides and hydroxyl groups of the polysaccharides [137]. The fungal cell has been observed to be a site for the precipitation of heavy metals forming NPs, as was seen by Mukherjee et al. [20], where Ag NPs were synthesised by the fungus *Verticillium* sp.

### 3.3. Bioaccumulation and Compartmentation

**Figure 4:**
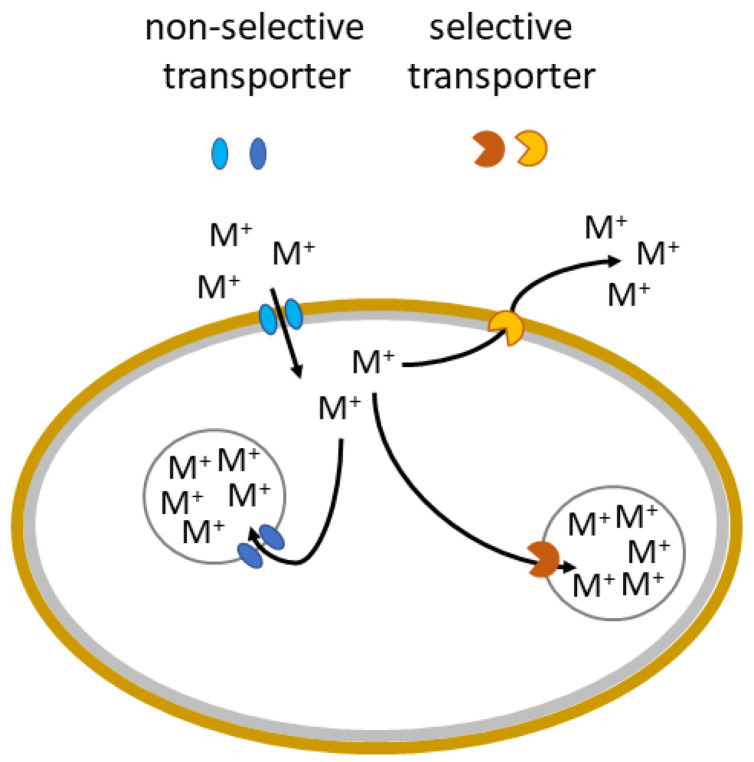
Bioaccumulation, compartmentation, and efflux of metals in fungi.

Passive sorption is just one of the strategies by which fungi resist metal toxicity. When a higher, toxic amount of metal ions enters a fungal cell, there are transport systems in place that help to reduce the damage by an accumulation of the metal in certain parts of the cell, most commonly vacuoles. Within these organelles, the metals are precipitated and can form NPs. This process of sequestering and precipitating metals in organelles of the cell is called compartmentation (Figure 4) and helps to reduce the toxicity of metals as well as creating a reserve of the metal for when the metal will have low availability from the external environment [26,93,134,138,139]. Therefore, increased concentrations of metals or toxic concentrations lead to vacuolisation in many species of fungi. Within these vacuoles, the biomineral formation of many metals occurs. Bioaccumulation and compartmentation are active processes requiring living cells that are thus dependent on the fungal metabolism. Since it is metabolically dependent, and there is a large variation in metabolisms of different taxa of fungi, the extent to which bioaccumulation and compartmentation are important for metal detoxification varies from species to species. In addition, bioaccumulation and compartmentation are dependent on environmental conditions, e.g., pH, and concentration of other elements and compounds [26,140]. Some studies suggest that bioaccumulation processes start after biosorption processes are no longer sufficient to keep metal concentration at optimal levels. It was reported that *Penicillium* sp. firstly uses biosorption and later also bioaccumulation as a response to toxic levels of Pb [98,141].

The compartmentation is heavily influenced by the types of proteins a fungus produces and, therefore, which metal ions it can easily compartmentalise. For example, the model organism yeast *S. cerevisiae* employs a defence mechanism against toxic effects of Mn via its trafficking to vacuoles. Transporters Ccc1 and Ypk9 are a part of membrane of vacuoles and they transport Mn from the cytosol to the inner environment of the vacuoles [93,142,143]. Such transporters were found for other metal ions in *S. cerevisiae* and also in a few cases for ascomycetes and basidiomycetes [93,102,144]. It was suggested that the ubiquitous aquatic ascomycete *Articulospora tetracladia* sequesters Ag ions into the vacuoles as a means of combating Ag toxicity [145]. 

It was suggested that Cd, Ni, and Pb were most actively bioaccumulated into vacuoles by the basidiomycete *Phlebia brevispora* [146]. Similarly, metal tolerance to Zn in the basidiomycete *Suillus bovinus* is based on its compartmentalisation into the vacuoles [147]. *Paxillus involutus* was found to rely on biosorption followed by bioaccumulation of Cd in vacuoles via Ca2þ ionophore A23187, a metabolically dependent process [148]. In the vacuoles of *P. involutus*, Cd is bound to sulphur-bearing components [104]. The intracellular accumulation of Zn by *Penicillium* sp. occurred probably via precipitation with polyphosphates [141].

### 3.4. Efflux of Metals

The efflux of metals is a vital part of the homeostatic systems of ions that control the import, storage, export, and transport within fungal cells [93,149]. While the more specific transport processes function during typical conditions, higher, potentially toxic concentrations of metals activate specific protection systems in fungi that assist in the exclusion of the metals [26,150]. Metal ion efflux often occurs through transmembrane channels—proteins that protrude from both sides of the membrane (Figure 4) [151].

In fungi, such efflux mechanisms were reported for As-induced toxicity in different strains of *Aspergillus* sp. [102,152]. *Hymenoscyphus ericae* collected from a mine site also demonstrated an enhanced As efflux, which suggests that it is a common resistance mechanism among different species of fungi [153]. A similar process was observed in the Zn-resistant strain of the basidiomycete *Suillus bovinus*, where a higher efflux was observed compared to non-resistant strains [147]. Resistance to Cu in the pathogenic fungus *Fusarium graminearum* is also based partly on efflux mechanisms to prevent host-enacted Cu toxicity [151]. In the case of Mn, a special transporter Pmr1, which was also characterised as an Mn^2+^ Golgi lumen transporter, serves in the efflux of Mn^2+^ from *S. cerevisiae*. Excess, potentially toxic Mn ions are transported to secretory vesicles, which are then released from the cell [154]. Another transporter that helps to excrete Mn ions from cells, Hip1p was found in *S. cerevisiae*. However, the transporter does not have an exact efflux path described as of yet [155]. For filamentous fungi, a transporter PcMnt was found in *Phanerochaete chrysosporium*, which has an important role in the homeostasis of Mn and is likely responsible for the release of Mn from the cell [156]. In the case of non-essential metals, efflux is often carried out by transporters of essential metals with similar properties. Such a process was observed in *Aspergillus nidulans*, where Cu exporter crpA was responsible for Ag export and resistance of the fungus [157].

## 4. Fungal Synthesis of Metal-Containing Nanoparticles

**Figure 5:**
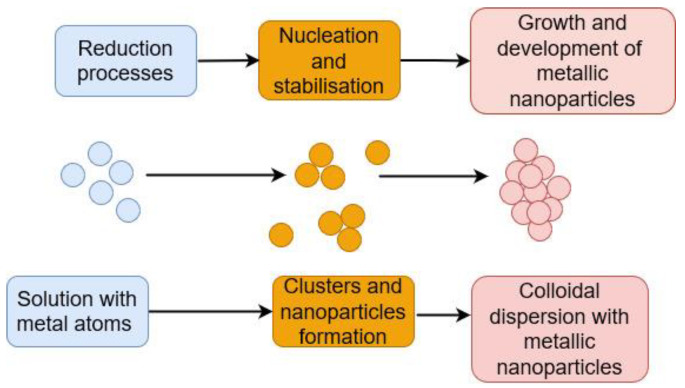
Classical bottom-up type of synthesis—the creation of metal NPs via chemical reduction.

**Figure 6:**
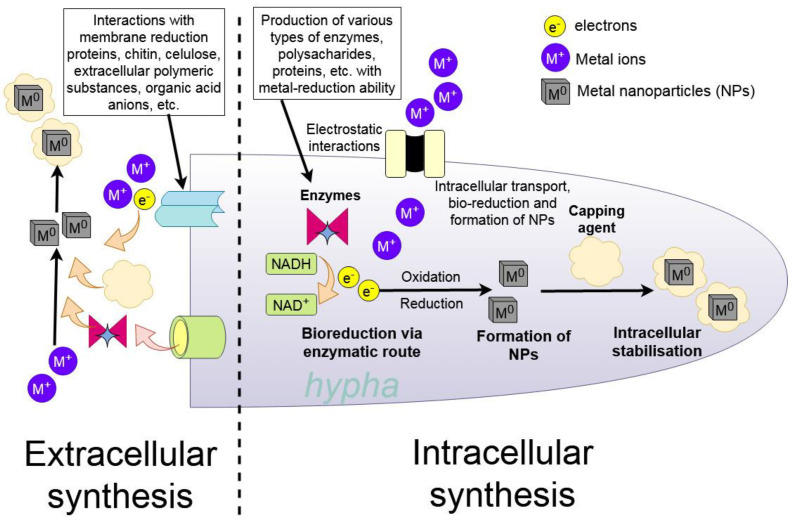
Mycosynthesis—the creation of metal NPs via the interaction with fungus and its metabolites.

NPs are often produced and stabilised using one of two approaches: top-down or bottom-up [158]. The bulk material is divided into small particles (size reduction) utilising various physical (grinding, milling, and thermal ablation) and chemical processes in the top-down strategic approach [159]. In the bottom-up approach, NPs are synthesised through the self-assembly of atoms into nuclei, which then transform into tiny particles. Chemical (electrochemistry, chemical, and photochemical reduction) and biological techniques of NP generation belong to this approach [160]. Figure 5 shows a synthesis of metal NPs from a metal precursor with the classical bottom-up system in comparison to the mycosynthesis of NPs generating biogenic nanominerals with the help of the filamentous fungus *A. niger* (Figure 6).

When NPs are formed by bottom-up synthesis, their stability in colloidal solutions depends on the surface charge. The NP charge is affected by several factors, e.g., the chemical composition of NPs, the type of interface, the presence of other ions in the solution, pH or the influence of external electromagnetic forces [161]. The properties of the interface between the two phases are theoretically determined by the different spatial organization of the charge that generates the electric double layer. The electric charge on the surface of the solid phase of NPs can be created by various mechanisms [162].

The use of biogenic synthesis of NPs has a number of advantages over traditional physical and chemical methods. Physical processes are more capital-demanding, requiring a high quantity of energy with lower production yields [160]. Using traditional chemical procedures, on the other hand, is costly and necessitates the use of harmful chemical compounds or organic solvents as reducing agents [163]. For the reason that hazardous compounds are used on the surface of NPs and non-polar diluents are used in the chemical production procedure, their use in clinical and biological domains is limited [164]. Biological NP synthesis can be carried out using organisms such as bacteria, fungi, and plants, as well as their metabolic by-products that act as reducing and stabilising agents. Because of the process of coating with biogenic surfactants or capping agents, NPs generated by organisms are clean, sustainable, stable, and often biocompatible [165]. They also have fewer environmental repercussions, differ in shape and size, and sometimes may be synthesised at a faster rate [164]. The biological capacity to act as a catalyst for processes in aqueous fluids at standard temperature and pressure settings accounts for the quicker pace of synthesis [163]. This technology is environmentally safe and free of pollutants, making it ideal for biological applications where purity is important [166]. Fungi may easily be grown on a large scale (“nanofactories”) and produce NPs with precise size and morphology [167]. Below, we have attempted to provide an overview of the most recent published research on the use of fungi for biogenic NP synthesis and subsequent applications. The mechanisms of synthesis are examined, and approaches for optimising the processes and the significance of NP capping are also discussed.

### 4.1. Mechanisms of Synthesis

Many research articles on the biogenic synthesis of NPs utilising fungi have been published. However, the specific mechanisms involved have yet to be thoroughly explored. In the case of NPs made of pure metal, such as Au or Ag, enzymes present in the fungal filtrate were observed to reduce the oxidation state of metal ions, creating elemental metal (M^0^) on a nanometric scale via the extracellular creation of NPs. For PbS NPs a two-step process was suggested [168]. The first step is the chelation of metal ions by phytochelatins, with the second step being S^2−^ generation that involves enzymes in the pu-rine biosynthesis pathway, where the phytochelatin–metal complex is transformed into a phytochelatin–metal sulphide complex. The colour of the filtrate usually changed after the reaction, and UV-visible spectroscopy was utilised to observe surface plasmon resonance bands that indicated changes in the material’s optical characteristics [169]. The existence of bigger NPs is indicated by the absorbance wavelengths of the generated bands and an absorbance peak at a longer wavelength [170]. The size of NPs is mostly determined by the synthesis circumstances, which include fungal species, temperature, pH, and a dispersion medium. Similarly, other NPs, such brown CdO NPs, have their typical colouration, which indicates the synthesis of NPs.

Many biomolecules can interact with metal ions to form complex electron transport pathways, such as those involved in the conversion of NADPH/NADH to NADP^+^/NAD^+^ [171]. The most important components in the biogenic synthesis of metal-containing NPs are nicotinamide adenine dinucleotide (NADH) and NADH-dependent nitrate reductase enzymes, both of which will be discussed in greater depth later. Due to the ease of downstream processing and biomass treatments, as well as higher productivity than bacteria, fungal production of NPs is a simple and easy technique.

For example, Metuku et al. [172] discovered that by extracellular biomineral formation of Ag ions, *Schizophyllum radiatum* (white rot fungus) could produce well-dispersed stable Ag NPs (size 10 to 40 nm). These NPs were found to have potent antibacterial action against a variety of Gram-negative and Gram-positive bacteria. The main benefit of extracellular synthesis of nanoscale material is that it is free of contaminants such as intracellular proteins and does not require ultrasonic treatment with detergents. Aside from that, comprehending the mechanistic features of NP manufacturing has become critical for designing trustworthy applications. Rajput et al. [173] investigated several *Fusarium oxysporum* fungal strains for Ag NP synthesis and investigated the effect of isolate selection, temperature, and pH on NP morphology in order to close this knowledge gap. According to their findings, understanding the interactions between the organic and interfacial layers will aid in the development of novel applications, notably in the field of biosensors.

To give examples of other NPs, Kitching et al. [174] identified *Rhizopus oryzae* cell surface proteins for in vitro synthesis of Au NPs for biomedical and biocatalytic applications. Suryavanshi et al. [175] used *Colletotrichum* sp. in the production of Al_2_O_3_ NPs, which were then functionalised with essential oils from *Eucalyptus globulus* and *Citrus medica*. Seshadri et al. [168] discovered that the marine yeast *Rhodosporidium diobovatum* has an advantage over terrestrial yeasts. The marine yeast synthesised PbS NPs even when Pb^2+^ was added simultaneously to the inoculum as opposed to the addition of metal ions in the mid-log phase of growth. Therefore, using marine yeast eliminated the necessity of growth-phase monitoring and is thus the preferred species for the synthesis of PbS NPs. These are just examples of the synthesis utilising different species of fungi. A more thorough explanation of factors that are important for NP mycosynthesis is given in the following subsections.

### 4.2. Factors Affecting Nanoparticle Synthesis

**Figure 7:**
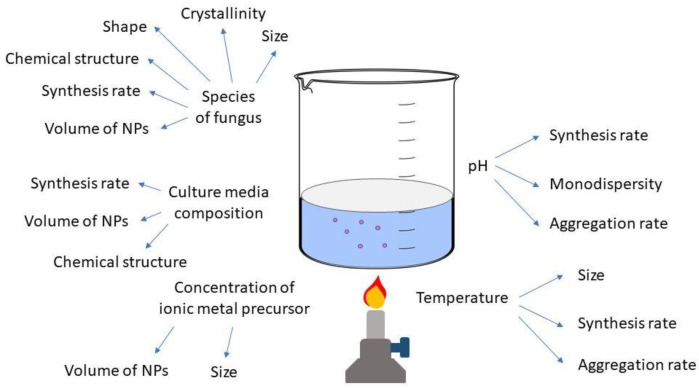
Factors affecting NP synthesis.

Given the large range of fungi that can be used in NP synthesis, it is critical to think about their specific features and adjust the synthesis settings accordingly [176]. As seen in Figure 7, temperature, agitation, metal precursor concentration, pH, light, culture media, biomass amount, and the fungus utilised all produce NPs with variable physicochemical properties [177].

#### 4.2.1. Temperature

The temperature employed in the mycosynthesis of NPs can have an impact on such characteristics as synthesis speed, NP size, and stability [170]. Azmath et al. [178] discovered that when NPs were synthesised using the endophytic fungus *Colletotrichum* sp., the reaction rate increased at higher temperatures, with the synthesis taking less than 20 minutes at higher temperatures. However, employing *Rhizopus stolonifer* filtrate at 80 or 10 °C, AbdelRahim et al. [179] reported no synthesis of Ag NPs, which they attributed to the denaturation or inactivation of enzymes and other components. Although most studies have found that higher temperatures result in faster rates of synthesis, the quality of the NPs must also be addressed. In addition to impacting the kinetics of synthesis, the temperature can change the final size and stability of NPs. Shahzad et al. [180] used the fungus *Aspergillus fumigatus* to make particles, agglomerates of which had a diameter of 322.8 nm at 25 °C and grew in size as the temperature increased, reaching a diameter of 1073.45 nm at 55 °C. Husseiny et al. [181] employed the fungus *Fusarium oxysporum* and discovered that as the temperature was raised to 50 °C, the NP size was reduced, with the smallest (30.24 nm) occurring at this temperature. The effects of temperature on the size and stability of the NPs vary depending on the fungus species utilised, as shown above. The ability of some fungal species to synthesise NPs at high temperatures suggests that electrons can be transported from free amino acids to Ag ions. However, very high temperatures, between 80 °C and 100 °C, cause the proteins that make up the NP capping to denature. The nucleation of M^+^ ions is altered by this denaturation, resulting in NP aggregation and growth in size [182]. High temperatures cause an increase in NP size and loss of stability due to the decreased activity of the enzymes involved in the production. In the synthesis of Au NPs [183], where plant pathogen *Macrophomina phaseolina* was used, the temperature was varied from 28 °C to 55 °C. The optimal temperature for the synthesis was 37 °C, where the largest number of Au NPs was produced. At higher temperatures of 45 °C and 55 °C, it was suggested that inactivation of the enzymatic processes occurs. Bi_2_O_3_ NPs were produced via extracellular synthesis utilising the plant-pathogenic fungus *F. oxysporum*. The synthesis was performed at a room temperature of 28 °C. Azam et al. [184] used *Penicillium oxalicum* in the synthesis of CdO NPs. The temperature they used was 30 °C. A temperature of 37 °C was also used in the synthesis of CdS NPs utilising extracellular metabolites of white rot fungus *Phanerochaete chrysosporium*. Meanwhile, a lower temperature of 26 °C was selected for in vivo synthesis of CdS NPs with white rot fungus *Pleurotus ostreatus* to provide the best growth conditions [185]. *Aspergillus nidulans* was used in the synthesis of Co_2_O_3_ NPs, where a temperature of 30 °C was used [186]. Cell-free extract of *Trichoderma asperellum* was used to synthesise CuO NPs [187]. The temperature was kept at 40 °C. *Aspergillus niger* isolated from mangroves was used in the synthesis of iron oxide, magnetite, and Fe_3_O_4_ NPs [188]. Film-form biomass of *Aspergillus aculeatus* was used to create NiO NPs [189]. From a temperature range of 20 °C to 60 °C, 30 °C was selected as optimal for the synthesis. Mycelia of *Trichoderma* sp. were utilised in the synthesis of PbSe NPs [190]. The selected temperature for the reaction was 30 °C. White rot fungus *P. chrysosporium* was also used to synthesise Pd NPs and the temperature used was 37 °C [191]. A temperature of 37 °C was also used in the synthesis of TiO_2_ NPs by *Aspergillus flavus* [192]. The temperature of the synthesis is selected mainly for the best growth conditions in the case of NP synthesis with living mycelia or it can be adjusted for the greatest yields or preferred size of NPs in the synthesis with extracellular metabolites or extracts of fungi.

#### 4.2.2. pH

Certain features of NPs can be controlled by adjusting the synthesis pH. According to Nayak et al. [193], the conformation of nitrate reductase enzymes can change depending on the quantity of protons in the reaction media, resulting in changes in the morphology and size of the NPs. There is more competition between protons and metal ions for forming bonds with negatively charged areas at higher pH, leading to increased synthesis success at alkaline pH. A higher alkaline pH resulted in a faster synthesis time as well as decreased NP size dispersion and polydispersity index values, according to Du et al. [194]. These characteristics suggest better stability due to the electrostatic repulsion of anions present in the dispersion [195]. *Colleotrichum* sp. synthesis of Ag NP was finished in roughly 20 minutes at an alkaline pH and a higher temperature of 50 °C than at a lower pH [178]. Successful syntheses of Ag NPs have been reported in certain investigations at neutral or slightly alkaline pH. No colour change was detected in synthesis employing *Guignardia mangiferae* between pH 1 and 4, although colouration began to develop at pH 5 and 6. The intensity of the dispersion increased when the pH was raised, with the NPs exhibiting stronger monodispersion and stability at pH 7 [196].

Similarly, pH has an effect on the synthesis of other NPs, and each type of NP has its optimal pH for synthesis. In synthesis utilising the plant pathogen *M. phaseolina* [183], Au NP production was the highest at pH 8, followed by pH 7. The most important reported effect of the reaction pH was on the ability to modify the charge on the various biomolecules involved in the formation and capping of the Au NPs. Optimal reaction pH was studied for NiO NPs created via biofilms of *A. aculeatus* [189]. Out of the pH range of 2 to 6, pH 4 was selected as the optimum for mycosynthesis. It was found that the optimal pH for the synthesis of PbSe NPs utilising *Trichoderma* sp. was pH 8 from a range of 5 to 9. At pH 5, no synthesis occurred, and at pH 9, only Se NPs were synthesised [190].

#### 4.2.3. Culture Media and Growth Parameters

Fungi are known to respond differently depending on the culture media and growing circumstances. Various metabolites and proteins are produced as a result of changes in these conditions [197]. In NP synthesis using fungi, a growth media containing substrate specific for the enzymes that function in the synthesis can encourage their development and release, enhancing the reduction of silver and the formation of NPs [181]. In investigations involving the growing of fungus on various media, distinct behaviours were discovered. Using *Sclerotinia sclerotiorum* grown in various broths, Saxena et al. [198] produced Ag NPs, with the maximum NP production achieved using a potato dextrose medium. Silva et al. [197] used the fungus *Duddingyonia flagans* to synthesise Ag NPs and then transferred the biomass to pure water and water containing insect carapaces as a natural source of chitin (a substrate for fungal enzymes). The filtrate treated with chitin had approximately three times the protein and produced more NPs. The amount of biomass utilised in the process can have an impact on the synthesis and properties of NPs. Some research revealed that a lower amount of biomass resulted in increased NP formation, while higher biomass concentrations resulted in higher synthesis rates [170]. Shahzad et al. [180] investigated the usage of 1, 4, 7, and 10 g of *Aspergillus fumigatus* biomass, finding that when 7 g of biomass was used, the NPs produced more, were smaller, and had better dispersion. Rose et al. [199] used *Penicillium oxalicum* and found that a more biomass resulted in higher production of NPs with more uniform size distribution, which they attributed to the mycelium’s increased release of the nitrate reductase enzyme. For NiO NP biofilm production [189], it was found that the second-highest amount of biomass, 1 g, from a range of 0.25 to 1.25 g was optimal for fungal synthesis. Synthesis of PbSe NPs [190] was carried out at different concentrations of biomass: 0.1, 0.3, 0.5, and 1.0 g. The optimal concentration of 0.5 g was selected, as it produced the highest number of NPs.

#### 4.2.4. Metal Precursors

A metal precursor was used at a concentration of 1 mM in a majority of the investigations using fungus for extracellular NP production [190,200]. A lower metal precursor concentration resulted in smaller NPs and better dispersion in some circumstances [201]. Using the fungus *Rhizopus stolonifer*, AbdelRahim et al. [179] obtained the smallest NPs (2 nm) at 10 mM AgNO_3_, while sizes of 54 and 14 nm were obtained at 100 and 1 mM, respectively. Previous research suggests that the concentration of metal precursors that are utilised to produce NPs with acceptable physicochemical properties has an upper limit. High concentration of metal ions results in very large NPs with uneven shape due to competition between the silver ions and functional groups from the fungal filtrate, as well as toxicity [180]. In addition, for the synthesis of some metal-containing NPs, the right precursor needs to be selected with attention paid to the state and coordination of the metal, which might undergo transformations upon reaction with the components of the synthesis media. In the case of Pd NPs produced using *P. chrysosporium* [191], the use of Pd^2+^ in form of Pd(NO_3_)_2_ was accompanied by the hydrolysis upon addition to the synthesis medium, which caused unwanted precipitation of insoluble Pd hydroxy species. Instead, the Pd^4+^ precursor Cl_6_K_2_Pd was successfully used to synthesise the Pd NPs.

#### 4.2.5. Biomolecules

Biomolecules have recently attracted attention due to their atoxic properties in the synthesis of homogeneous NPs. The NPs produced by mycosynthesis via biomolecules can differ from their equivalents created by chemical or physical methods. The difference can be noticeable, for example, in the case of colloidal dispersions with a naturally occurring biomolecule coating, which can facilitate the adsorption of NPs on the surfaces and active sites of target substrates and can also ensure their higher stability in aqueous phases [202]. For the formation of NPs for one functional biomolecule, monodisperse NPs (of uniform crystallinity, size and shape) can be formed, but if a microscopic fungus excretes several types of metabolites, polydisperse NPs can be formed [14]. The beginning of the process induces the formation of a nucleus (nucleation), which is formed either from a homogeneous saturated true solution (homogeneous nucleation) or in the presence of other components with active surfaces (heterogeneous nucleation) [203].

For metal-containing NPs, their surface coating is also important, as it determines the thermodynamic stability of the particles in the solution. The variability of functional groups in the structures forming the envelopes of biogenically formed NPs causes differences in their solubility and reactivity [204]. When evaluating the impact of biogenic coatings on NPs in a colloidal suspension, the geometry of the molecules forming this coating, their rotation and their ability to bind into larger spatial formations must be taken into account, e.g., by chaining, branching, formation of rings, dendrimers, etc. Their character plays an important role in predicting the behaviour of NPs in the colloidal system or their degradation. The molecular and physico-chemical aspect of biogenic NP packaging determines the application potential of NPs and their fate in the environment. Several types of molecules were observed to be active in the formation and stabilisation of mycosynthesised NPs (Table 1).

Amino acids are one type of biomolecule that can be used to adjust NPs with specific structures by acting as reducing and capping agents. Using L-histidine amino acid as capping agent, researchers created Au NPs (by reducing tetrachloroauric acid) with a size range of 4–7 nm. The amino acid content was shown to have an inverse effect on the size of NPs, and the amino and carboxyl groups in the amino acids were found to be responsible for the reduction in bulk salt and NP surface coating [211]. Some investigations on the fungal synthesis of NPs reveal that unbound amine groups or cysteine residues interact electrostatically with the NP surface [209]. Gade et al. [205] used elemental spectroscopic imaging to establish the presence of sulphur atoms around Ag NPs produced with *A. niger*. In this case, the NP was stabilised by native protein molecules binding to its surface via sulphur atoms in their constituent amino acids. In a related study, glutamic acid and histidine amino acids were used to make Au nanochains in a single step in less than 15 minutes [212]. Indication of amino acids was also observed in TiO_2_ NPs that had fungal capping made of biomolecules of *Aspergillus flavus* [192].

Ligand capping agents have a crucial and flexible role in the functionalisation and stabilisation of NP synthesis. The agents can be utilised to add valuable features to NPs by managing their morphology, desired form, and size, as well as protecting the surface against aggregation [213]. Commercial surfactants can be used as capping agents, but they are non-biodegradable and dangerous to the environment, necessitating the employment of environmentally friendly capping agents. Similarly, enzymes, peptides, and proteins are important reducing and capping agents in fungi [214]. The responsibilities of several sorts of prospective capping agents (that could act or be utilised) have been discussed further.

Polysaccharides are a form of polymeric carbohydrate molecule that consists of repeated mono- or disaccharide units joined together by glycosidic connections that can serve as capping agents. They are low-cost, hydrophilic, stable, safe, and biodegradable, and atoxic NPs can be made with them when water is the solvent [215]. Polysaccharides are known for their ability to significantly enhance the kinetics of sol–gel processes due to their catalytic action [216]. Chitin, together with the fungal proteins with amino groups, was indicated to be responsible for the synthesis of Pd NPs using *P. chrysosporium*. The palladium cations were probably coordinated by the acetylamino group of the chitin moieties. Chitosan is a molecule derived from the deacetylation of chitin and is produced either from the shells of crustaceans or from fungal cell walls of fungi [217]. It is a natural preservative and has antimicrobial properties [218]. Chitosan has a perspective in tissue engineering due to its biocompatibility, biodegradation, and osteoconductivity [219]. Another potential avenue, where chitosan plays a role, is an agrobiotechnological application, where it is used as an antifungal agent and gene modulator [220]. In addition, it can form the coating of the mycosynthesised nanoparticles, limiting their size. Endophytic and pathogenic fungi naturally produce chitosan to evade the natural defences of plants and thus could be used in the mycosynthesis of NPs that have a coating made of chitosan, or the chitosan could be generated by fungi and later added to the synthesis process [220,221]. Dextran is a branching polysaccharide, with chains of different lengths, that is used to coat metal NPs. It is complex, hydrophilic, biocompatible, and atoxic [222]. Natural honey was used to produce Au NPs (spherical, 15 nm) in water, acting as a reducing and protective agent. The honey’s fructose was expected to act as a reducing agent, while proteins were responsible for the NPs’ stability [210]. On fungal ZnO NPs, Kadam et al. (2019) discovered capping proteins with molecular weights of 52 and 58 kDa. Similarly, on fungus-produced magnetite NPs, Bharde et al. [207] found two capping proteins of 55 and 13 kDa. Rajakumar et al. [192] also indicated amino fungal proteins as capping agents when TiO_2_ NPs were synthesised with *A. flavus*. Similarly, protein capping acted to stabilise Co_2_O_3_ NPs synthesised with *A. nidulans* [186] and protein residues such as cysteine and methionine were suggested as important parts of the surface capping of the NPs. Because the surface functionalisation of capping proteins dictates the majority of NP features, understanding them can aid in the development of specialised applications for fungus-mediated NPs. Aminocellulose is a cellulose backbone-linked aminooxy derivative with a nitrogen functional group. In another study, Ag NPs (2 to 14 nm) were produced utilising amino cellulose as a reducing and capping agent, with cellulose reducing ions to Ag(0) at high temperatures [223]. As a result, these environmentally benign capping agents can be employed as renewable green alternatives to harmful chemicals in the manufacture of NPs, preserving the environment.

Fungi synthesise NPs through a variety of bioreduction and other mechanisms involving proteins and extracellular or intracellular enzymes. Several fungi have been shown to produce extracellular enzymes that act as reducing agents in the formation of NPs. Extracellular enzymes such as acetyl xylan esterase, cellobiohydrolase D, glucosidase, and beta-glucosidase are examples [163]. Nitrate reductase, a NADH-dependent reductase enzyme secreted by fungi, e.g., *Fusarium oxysporum,* is involved in the bioreduction (M^+^ ions to M^0^) and extracellular synthesis of metal NPs, i.e., Ag and CdSe NPs [208,224]. In addition, nitrate reductase from *F. oxysporum* was successfully employed to synthesis NPs in an in vitro investigation in the absence of oxygen in the presence of NADPH (cofactor), phytochelatin (stabiliser protein), and 4-hydroxyquinoline (electron carrier). When *A. niger* and *A. fumigatus* were treated in an AgNO_3_ solution, where fungal proteins act as stabilisers, they formed Ag NPs extracellularly much faster than other procedures [225]. The liberation of NADH-reliant enzyme nitrate reductases by *Penicillium fellutanum* and *P. brevicompactum* has also been found to cause fast metal ion reduction [226]. *Cladosporium cladosporioides* and *Coriolus versicolor* were also used in the extracellular production of Ag NPs, which involves fungal proteins, organic acids, and polysaccharides, all of which affect the development and morphology of the nanocrystals [227]. According to previous findings, certain proteins may be responsible for the reduction of M^+^, resulting in the creation of NPs [228].

Because metal ions diffuse across the membranes, NPs are generated as a result of enzymatic reduction accumulated in the periplasmic space, the cytoplasmic membrane, and the cell wall of the fungi. This is the intracellular enzyme mechanism of metal bioreduction, in which fungal cells and sugar molecules both play important roles. The gathering of metal ions from the medium and subsequent reduction inside the fungal cell is said to rely on the interactions of internal enzymes and positively charged groups [229]. *Verticillium* was exposed to Ag^+^ and Au^+^ ionic solutions, which caused intracellular reduction and the production of Ag and Au NPs, respectively. Additionally, electron imaging indicated that a toxic NPs were produced within the cell wall as a result of enzymatic bio-reduction by reductase enzymes [20,25]. Incubation of *Phanerochaete chrysosporium* in an ionic Au^3+^ solution resulted in the production of Au NPs with sizes ranging from 10 to 100 nm. Laccase was utilised as an extracellular reducing agent, while ligninase was discovered to be responsible for Au^3+^ ion reduction within the cell [209]. The form of NPs is influenced by the fungi’s incubation period, the concentration of the metal salt solution, and the reaction mixture’s incubation conditions. In the case of the marine yeast *R. diobovatum*, phytochelatins were identified as important for the formation of PbS NPs [168]. Metal ions are chelated by phytochelatins, and in the following step is S^2-^ generated by enzymes in the purine biosynthesis pathway, where the phytochelatin–metal complex is transformed into a phytochelatin–metal sulphide complex. Phytochelatins and other peptides also attach to the surface of the nanoparticles, providing capping that hinders further growth.

During the formation of NPs, electrons can be transported by low-molecular-weight redox mediators such as ubiquinol, NADH, or oxygen/superoxide, or by direct interaction between c-type cytochromes redox proteins and the metal ion [230]. However, the ability to produce Ag NPs in *F. oxysporum* strains varies depending on the reductase/electron shuttle interactions under these conditions. In addition to these extracellular enzymes, Durán et al. [30] discovered many naphthoquinones and anthraquinones in *F. oxysporum* that have outstanding redox characteristics and could serve as electron shuttles in metal reductions. Hydroxyl groups and polyphenols were indicated to be responsible for the synthesis of ZnO NPs by the fungus *Xylaria acuta* [231].

For the last two decades, fungal exopolysaccharides (EPSs) have been recognised as high-value biomacromolecules. Pullulan, scleroglucan, and botryosphaeran, for example, have a variety of uses in chemical industries, pharmaceuticals, medicine, and food. Over the last two decades, EPS generation by fungi has been thoroughly investigated. EPS synthesis by fungi such as *Ganoderma lucidum, Agaricus blazi, Cordyceps sp., Lentinus edodes*, and *Grifola frondosa* has been observed in submerged cultures, all of which exhibit different and fascinating biological activity [232]. Despite the importance of fungal EPSs, current knowledge of fungal biosynthesis is limited, and a comprehensive search for new fungal species capable of producing novel EPSs is still required. Most of the time, the molecular weight variations and sugar compositions of fungal EPSs are influenced by the culture medium composition and the physical circumstances provided during fermentation. An endophytic *F. oxysporium* has been identified as an EPS generator. Furthermore, electrostatic interactions between cationic metal ions and anionic groups such as the carboxylic and phosphoric functional groups of EPS have been cited as a benefit for metal NP production [233]. Hydroxyl, carboxyl, phosphoric, hemiacetal, and amino end groups have all been proposed as ways to reduce metal ions from precursor salts to create the desired NPs [234].

Different species of fungi produce various molecules at differing quantities and thus may produce differently shaped and sized metal-containing NPs [235]. Mycosynthesis of ZnO NPs was compared in two species of fungi—*Fusarium keratoplasticum* and *A. niger*. Aqueous extract of *F. keratoplasticum* was used to synthesise hexagonal ZnO NPs, while *A. niger* extract produced rode-shaped NPs.

### 4.3. Intracellular and Extracellular Synthesis

**Figure 8:**
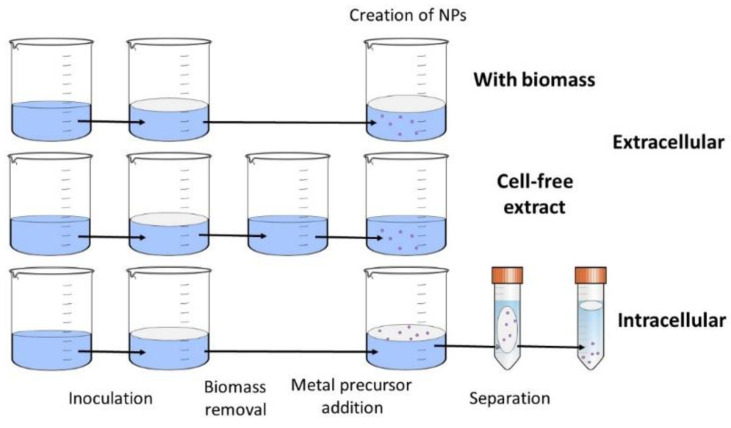
Intracellular and extracellular synthesis of nanoparticles by fungi.

Metal-containing NPs were produced by fungi through two different pathways intracellular synthesis inside the fungal cells, which requires an additional extraction step, and extracellular synthesis, which encompasses both synthesis in mycelial growth media in the presence of fungi and also with cell-free fungal extracts, which use the plethora of fungal biomolecules to mineralise and stabilise the created NPs (Figure 8).

The intracellular enzymes responsible for NP synthesis in fungi are hydrogenases and cellular ATPases. Fungal antioxidants are also responsible for NPs precipitation as a defensive mechanism from the toxic metal ions in the environment. The intracellular extract is usually prepared by physical or chemical disruption of the mushroom cell to release active molecules, such as cytoplasmatic reductases [236]. Partially intracellular NPs can be synthesised by the defence mechanism of mushroom cells against the actions of metal ions. Antioxidants in these processes reduce part of these metal ions into NPs inside the cell [237].

A majority of mushrooms produce extracellular proteins, polysaccharides, quinones, peptides, oxidoreductases, and other molecules as a result of their metabolism and/or defence mechanism against unbalanced environmental conditions, such as a high concentration of metal ions. The metal ions are reduced through the presence of these organic molecules and stabilised via precipitation, aggregation, biocoupling or biosorption [236]. Extracellular reductases such as tyrosinases, Mn peroxidases, laccases and phenol oxidases play a major role in providing highly stable, water-soluble metal-containing NPs by reduction of the metal ions [238]. The advantage of extracellular secreted molecules is the easier separation from the biomass compared to intracellular, where the disruption of cells needs to be provided [239].

## 5. Future Perspectives and Conclusions

Although studies published to date show that many different species of fungi can produce biomolecules necessary for the formation of various metal-containing NPs, optimisation of these processes will be needed for scaling up to commercial production, and the selection of the species may be of great interest for some specific applications, such as in biomedicine, where fungi may not only form and stabilise the NPs but also provide other biomolecules that have important properties in their application, e.g., pharmaceutical effects of medicinal mushrooms or antimicrobial effects of certain molecules that such fungi as *Penicillium* sp. produce. These kinds of synergies of the inorganic core with organic capping are an avenue for future research.

It is obvious that diverse synthesis circumstances might result in different NP features, as well as synthesis success or failure. The impacts of the many parameters, on the other hand, are unclear, necessitating further extensive investigations for each fungus used. It is also crucial to define the needed physicochemical properties of the NPs in order to set the parameters used in the synthesis, such as temperature, pH, and time. The optimisation of synthesis techniques should allow for the rapid production of large quantities of NPs. This opens the door to using nanomaterials to overcome such problems as antibiotic resistance in bacteria and phytopathogens that harm agricultural production.

Furthering our knowledge in fungal synthesis will require more in-depth knowledge of the mechanisms of synthesis, where the whole process from nucleation with enzymes or on the biotemplates, and growth of the NPs to eventual capping by various biomolecules is described.

## Figures and Tables

**Table 1:** Selected examples of biomolecules used in the synthesis of various metal-containing NPs.

Species of Fungus	NP Type	Size (nm)	Biomolecule	Biomolecule’s Role	Source
*Aspergillus flavus*	TiO_2_	62 to 74	Fungal proteins, amino acids	surface capping	[192]
*Aspergillus niger*	Ag	20	nitrate reductase and anthraquinones	precursor reduction and NP formation	[205]
			fungal proteins	surface capping	
*Aspergillus terreus*	Co_3_O_4_ CuO Fe_3_O_4_ NiO ZnO	5 to 15 10 to 30 20 to 40 20 to 60 20 to 50	fungal proteins	precursor reduction and NP formation surface capping	[28]
Cs-HK1 fungus	Ag	10 to 30	exopolysaccharides	precursor reduction and NP formation	[206]
				surface capping	
*Fusarium oxysporum*	Fe_3_O_4_	20 to 50	20–30 kDa fungal proteins	hydrolysis of NP precursors	[207]
	CdSe	9 to 15	nitrate reductase protein/peptide	precursor reduction surface capping	[208]
*Phanerochaete chrysosporium*	Au	10 to 100	laccase	extracellular formation	[209]
			ligninase	intracellular formation	
			fungal proteins	surface capping	
	Pd	10 to 14	chitin, fungal proteins	precursor reduction, NP formation, and surface capping	[191]
*Rhodosporidium diobovatum*	PbS	2 to 5	peptides like phytochelatin	surface capping	[168]
			phytochelatinspurine biosynthesis pathway enzymes	intracellular formation	
*Trichoderma* sp.	PbSe	10 to 30	fungal proteinsreductase	Se reductionsurface cappingPbSe formation	[190]
*Volvariella volvacea*	Ag	15	fungal proteins	reducing agents	[210]
	Au	20 to 150		surface capping	
	Ag-Au	5 to 100			
